# Mitochondria: From Physiology to Pathology

**DOI:** 10.3390/life11090991

**Published:** 2021-09-21

**Authors:** Francesco Bruni

**Affiliations:** Department of Biosciences, Biotechnologies and Biopharmaceutics, University of Bari Aldo Moro, Via Orabona 4, 70125 Bari, Italy; francesco.bruni@uniba.it

Over the past decade, the role of mitochondria has extended beyond those tasks for which these organelles are historically known. Recent proteomics investigations have highlighted the extraordinary complexity of mitochondrial protein organization, which is a reflection of the numerous and disparate mitochondrial functions [1]. These include the synthesis of most of the ATP present in the cell, apoptosis, ion homeostasis, cellular stress response, antioxidant control, redox regulation, mitophagy, involvement in various biosynthetic pathways and many more processes. Furthermore, mitochondria are dynamic organelles, and their morphology can vary significantly within a cell. This is mainly due to the mitochondrial fusion and fission processes, referred as mitochondrial dynamics, which are crucial for the organelles’ interactions with other cell compartments and for the cross-talk with the cell cycle and metabolism as well as differentiation and senescence [2].

An intriguing feature of mitochondria is that they possess their own DNA (mtDNA), which is functionally coordinated with the nuclear genome. Indeed, numerous nucleus-encoded proteins are required for complex molecular processes such as replication, transcription, RNA processing and degradation, translation, and assembly of respiratory chain complexes that take place inside the mitochondria [3,4]. On the other hand, mitochondrial gene expression plays an important role in the communication between mitochondria and the nucleus, contributing to the regulation of cell physiology. Recent studies have pointed to novel RNA-driven mechanisms according to which mitochondrial-derived transcripts, in addition to their role in maintaining mitochondrial function, act as signalling molecules modulating the innate immune response [5]. 

Dysfunctions affecting the mitochondria can lead to various pathological conditions, including aging and neurodegenerative disorders, and are associated with a whole range of complex genetic disorders, known as mitochondrial diseases [6]. In recent years, great advances have been made in the field of mitochondrial diagnostics thanks to the rapid development of next-generation sequencing (NGS) technologies, particularly the whole-genome approach [7]. However, most of the aspects and mechanisms underlying mitochondrial diseases remain unclear and, to date, effective therapies are still lacking. Therefore, a thorough understanding of the molecular processes occurring in mitochondria is essential from a pathological perspective.

The Special Issue “Mitochondria: from Physiology to Pathology” published in *Life* (ISSN 2075-1729) collects a series of research and review articles and aims to provide an updated view of the main topics covering the physiological and the pathological aspects of mitochondrial biology.

Several contributions focus on the mitochondrial genome and its link to the physiopathological aspects of the various mitochondrial processes. Chapman et al. [8] present a comprehensive review on mtDNA metabolism, highlighting the interplay occurring between the organization of the mitochondrial genome, mitochondrial dynamics and cristae structure. After a brief excursus on the organization of mtDNA and on its main processes, such as replication and transcription, the authors deal in depth with the processes of fission and fusion relative to the structure of mtDNA, discussing the known genetic defects that affect mitochondrial dynamics. To complete the picture, they describe the close relationship between the nucleoid, OPA1 and the other “cristae-shaping proteins” constituting the MICOS complex, whose interactions modulate the formation and dynamics of mitochondrial cristae. These are critical process that, if not well regulated, can prompt mitochondrial dysfunction with the onset of neuromuscular diseases. Another important process whose dysregulation can lead to severe pathologies is the elimination of mitochondrial genome. Orishchenko and colleagues [9] review the mechanisms underlying the regulation of heteroplasmy level and mtDNA segregation, illustrating in detail the natural process of mitochondrial genome elimination in both the somatic cells and the germline. The development of methodologies for artificial manipulation of the mtDNA heteroplasmy level that aim either to prevent or potentially cure human diseases associated with mitochondrial dysfunction, is also examined.

Mitochondrial encephalopathy with lactic acidosis and stroke-like episodes (MELAS) is a metabolic disorder caused by point mutations in the mitochondrial tRNALeu^(UUR)^ gene, with a prevalence of A>G substitution in position 3243. Several papers have reported that overexpression of human mitochondrial leucyl-tRNA synthetase (LARS2) or its C-terminal domain (Cterm) has proven effective in rescuing the pathological phenotype in cellular models. In their research article, Capriglia et al. [10] investigated the molecular basis underlying the ability of the Cterm domain in rescuing the MELAS phenotype. The cellular model employed, consisting of a trans-mitochondrial cybrid line with a >95% mutation load, confirmed the therapeutic potential of the Cterm peptide but also showed that its rescuing ability was independent of the mitochondrial bioenergetics, unlike what has previously been observed in other cybrid lines. The authors proposed that the beneficial effect of this peptide could also be mediated by retrograde mitochondrial signals or, alternatively, by its potential ability to bind regulatory RNA in the cytosol. This research indicates that the full understanding of Cterm-rescuing mechanisms imposes the development of tissue-specific cellular models that accurately reproduce the pathological MELAS phenotype.

Another study, based on a cohort of 468 subjects from the Siberian region, was conducted by Kirichenko et al. [11] with the purpose of determining the impact of mitochondrial heteroplasmy measurements in the prognosis of atherosclerosis development. They investigated the association of nine different mtDNA mutations with carotid intima-media thickness (cIMT), a measurement of the artery wall thickness obtained by ultrasound imaging. Several mtDNA variants correlated with the mean cIMT, thus constituting potential prognostic markers. Interestingly, the mutations m.13513G>A and m.14846G>A showed a significant inverse correlation being associated with a low value of cIMT, representing good candidates for the development of anti-atherosclerotic gene therapies.

The dysregulation of mitochondrial functions certainly depends on faulty mitochondrial gene expression at different levels, but also on other factors. One of these, which takes on particular importance in mitochondrial physiology, is the post-translational modification of mitochondrial proteins. Phosphorylation is commonly employed in mitochondria either to modify protein functions or to activate fundamental signalling pathways, inside and outside the mitochondria. Kotrasová et al. [12] center their review on mitochondrial kinases and their role in keeping organelles fully efficient. This function is exerted on various and distinct substrates: mitochondrial import machinery, subunits of respiratory chain complexes and proteins involved in the main steps of mtDNA maintenance and expression. In addition, the activity of mitochondrial kinases is also important for organelle quality control and apoptosis. One of the central key players in mitochondrial quality control pathways is PINK1 (PTEN-induced kinase 1). Barroso Gonçalves and Morais [13] emphasize how valuable the supply of PINK1 is for the mitochondrial clearance through mitophagy and for the maintenance of mitochondrial homeostasis, thereby keeping cells healthy and functional. The precise mechanisms that mediate PINK1 function in the different mitochondrial pathways remain to be elucidated. Nevertheless, the genetic link between PINK1 and Parkinson’s Disease (PD) has long been proven, particularly for the familial form of this pathology. Further research needs to be carried out to define whether the mitochondrial homeostasis imbalance, due to the aberrant function of PINK1, is the key element that contributes to the pathological phenotype in both the familiar and sporadic forms of PD.

An important aspect of mitochondrial physiology is its hormone-mediated regulation. Medar et al. [14] analysed mitochondrial response to luteinizing hormone (LH) stimulation in Leydig cells, the major producers of steroid hormones that regulate sexual differentiation and development. In these cells, endocrine function requires the cAMP signalling pathway, whose involvement in modulating respiratory chain complexes activity, ATP production, biogenesis, import, dynamics, and mitochondrial-dependent apoptosis has been widely investigated. The reported results, obtained by different experimental approaches in rat Leydig cells ex vivo and in vivo with a different LH environment and steroidogenic capacity, supported the involvement of LH-cAMP pathway in the regulation of mitochondrial biogenesis and dynamics coupled with mitochondria-mediated steroidogenesis. Additional studies carried out in Leydig and other steroidogenic cells, e.g., derived from adrenal glands and placenta, will shed light on the pathogenic mechanisms triggered by the presence of unhealthy mitochondria in these tissues.

The last research article of this Special Issue focuses on the *Saccharomyces cerevisiae FAD1* gene, encoding the FAD synthase that adenylates the flavin mononucleotide (FMN) to flavin adenine dinucleotide (FAD), an essential coenzyme for various flavoenzymes. Barile and colleagues previously proved that FAD-forming activities, paralleled by FAD precursors uptake in mitochondria and mitochondrial FAD export to cytosol, could be specifically revealed in mitochondria. However, a protein responsible for the synthesis of FAD had never been identified in yeast mitochondria. In this paper [15], the presence of two Fad1p echoforms, dually localised to the cytosol and mitochondria, was reported. Intriguingly, the authors demonstrated the existence of two pools of *FAD1* mRNAs with 3’ untranslated regions (UTRs) of different length and containing a mitochondrial targeting signal. Therefore, the 3′ UTRs would be responsible for the fate of Fad1p echoforms, with the long *FAD1* mRNA generating the mitochondrial Fad1p. In this context, the authors discussed the role of specific RNA binding proteins (e.g., Puf3p) that modulate the import of the mitochondrial Fad1p echoform. Overall, the paper adds a useful piece of knowledge to the post-transcriptional control of genes encoding mitochondrial proteins, proposing the existence of novel regulatory mechanisms in yeast as well as in higher eukaryotes.
